# Information-Entropy-Guided Counterfactual Stability Modeling and Replay-Based Selective Override for Context-Fragile Branch Prediction

**DOI:** 10.3390/e28090977

**Published:** 2026-09-02

**Authors:** Yijie Cheng, Chengrui Yin, Yong Tan, Xiaofeng Yang

**Affiliations:** School of Electronic Engineering, Xi’an University of Posts and Telecommunications, Xi’an 710121, China; dal_cyj@stu.xupt.edu.cn (Y.C.); incherry@stu.xupt.edu.cn (C.Y.); ty_xian_you.dian@stu.xupt.edu.cn (Y.T.)

**Keywords:** information entropy, branch prediction, TAGE predictor, counterfactual reasoning, conditional entropy, selective override, microarchitecture, context fragility

## Abstract

Modern out-of-order processors depend critically on TAGE family branch predictors, which often struggle with context-fragile branches whose outcomes are highly sensitive to slight perturbations in the most recent global history. Existing predictors are confined to single-path factual reasoning and lack any mechanism to explicitly probe the local stability of a prediction. We formalise this limitation from an information entropy perspective: a context-fragile branch corresponds to a high conditional entropy region in the joint space of recent history bits and branch outcomes, where a single bit flip in the youngest history can shift the posterior branch probability across the decision boundary. To address this, we propose Mirror-TAGE, a lightweight microarchitectural framework that integrates counterfactual stability modeling with replay-guided selective overrides. Building on a TAGE-SC-L baseline following Seznec, Mirror-TAGE injects controlled bit-level perturbations into the youngest global history bits to construct two mirrored views, screened by an entropy-based reliability filter. We define a local prediction entropy, derive an information-theoretic characterisation of override correctness via DRT-filtered agreement, and decompose uncertainty into aleatoric and epistemic components. Under a paired causally consistent learning framework on synthetic workloads isolating the context-fragile regime, Mirror-TAGE achieves a 0.82 pp gain (66% override correctness, 7.5% overhead). Of successful overrides, 78% occur at entropy > 0.85 bits, consistent with the entropy-guided criterion, though partly driven by the entry gate design. Six SPEC CPU 2017 traces confirm no accuracy degradation, with gains of up to 0.38 pp. These results support entropy-guided counterfactual stability modelling as a promising branch prediction paradigm.

## 1. Introduction

Accurate conditional branch prediction is essential for sustaining high fetch bandwidth in deep out-of-order processors [1]. Modern predictors have evolved from simple saturating counters to history-rich mechanisms exploiting global, local, and path correlations [1,2,3], with the tagged geometric history length (TAGE) family [4,5] serving as the dominant reference point. Despite refinements, including mixed history usage [6], statistical correctors [5,7], and perceptron hybrids [8,9], residual mispredictions still leave substantial IPC headroom unrealised [10]. Hierarchical prediction structures [11] further confirm that this headroom persists even with expanded storage budgets. We observe that many residual errors share a common signature: the baseline predictor is not globally uninformed but locally fragile. From a Shannon-entropy perspective [12], these branches reside in high conditional entropy regions of the history-outcome space.

The information entropy perspective provides several analytical advantages. The conditional entropy $H(Y_{t}|h_{t})$ supplies a unified metric for context fragility that subsumes traditional confidence indicators such as counter saturation and alternate disagreement. The mutual information between mirrored views, $I(M_{t}^{\left(A\right)};M_{t}^{\left(B\right)}|h_{t})$, quantifies the structural stability of the local prediction landscape in bits, providing a principled threshold for intervention. Additionally, an entropy decomposition into aleatoric and epistemic components, representing irreducible branch randomness and reducible model uncertainty, respectively, enables the DRT to learn which entropy regime benefits from counterfactual correction and which does not.

The main contributions of this work are as follows:

1. We formally define and characterise the context-fragile branch regime from an information entropy perspective, showing that traditional single-path factual reasoning is inherently insufficient when the local conditional entropy exceeds a critical threshold (Section 2.3).
2. We propose a novel counterfactual stability mechanism that constructs two concurrent mirrored history paths by flipping either the most recent or the second most recent bit of the global history register, deriving a highly selective reliability estimate through entropy-inspired cross-view consensus and score margin criteria (Section 3.2).
3. We introduce a replay guided selective override framework managed by a Disagreement Replay Table that monitors and learns the historical usefulness of counterfactual corrections, ensuring that mirrored advice acts as a precision-targeted, low-frequency corrective filter (Section 3.4).
4. We develop an information-theoretic analysis comprising a local prediction entropy metric, a characterisation relating override correctness to the empirical agreement rate between the mirrored direction and the true outcome, and an aleatoric/epistemic entropy decomposition of prediction uncertainty (Section 4).
5. We implement a rigorous paired causally consistent learning evaluation framework and report consistent, statistically significant accuracy gains across multiple synthetic workloads, while comprehensive ablation studies, capacity scaling, and robustness sweeps provide evidence that explicit entropy-guided stability modelling is a practical pathway for extending modern predictors (Section 5). We complement the synthetic evaluation with a preliminary assessment on six SPEC CPU 2017 traces (Section 5.14) and discuss the path toward full validation in Section 6.

Table 1 positions the proposed framework against representative prior studies along several dimensions that are relevant to the specific design space explored in this work. Each prior predictor was designed with different objectives, and the absence of a feature does not imply a deficiency in that design. The comparison highlights the particular combination of techniques that Mirror-TAGE integrates and is not intended to suggest that all prior predictors should have pursued these specific directions, since each prior design targets different objectives within different constraints.

## 2. Background and Motivation

### 2.1. The TAGE Predictor Family

Composite confidence mechanisms [13] and entropy-based analysis of branch predictability [14] have further proved that the value of a predictor depends heavily on its ability to quantify uncertainty and selectively reverse weak baseline predictions. Neural and path-aware predictors, ranging from perceptrons [3] to convolutional neural branch predictors [15], piecewise linear designs [9], and multiperspective perceptrons [8], have demonstrated that mapping control flow history through broader feature spaces can capture correlations missed by conventional two-level tables. More recently, Schall et al. [11] introduced a last-level branch predictor that exploits a large backing store to capture hard-to-predict branches, illustrating the continued interest in targeted auxiliary structures for residual mispredictions.

More broadly, the trend toward low-complexity, targeted hardware co-optimisation is evident across modern microarchitectural design. Precisely scoped auxiliary structures can yield disproportionate efficiency gains, a principle that Mirror-TAGE exploits by confining its intervention to a narrow, entropy-guided counterfactual probe rather than scaling the primary prediction tables.

Despite these advances, all existing branch prediction architectures share a fundamental representational limitation: they interrogate the observed (factual) history captured at the fetch stage.

### 2.2. Information Entropy in Prediction Systems

For a binary branch outcome $Y_{t}\in \left\{0,1\right\}$ with probability $p=P(Y_{t}=1|h_{t})$, the conditional entropy is

(1) $$H\left(Y_{t}|h_{t}\right)=-p\log_{2}p-\left(1-p\right)\log_{2}\left(1-p\right).$$

The mutual information between the history and the branch outcome, $I\left(Y_{t};h_{t}\right)=H\left(Y_{t}\right)-H\left(Y_{t}|h_{t}\right)$, quantifies how much predictive information the history provides about the outcome [12,16]. A high mutual information indicates that the history is informative, whereas a low value indicates that the history provides little beyond the marginal rate.

In the context of branch prediction, Chen, Coffey, and Mudge [14] first applied entropy concepts to analyse branch predictability. Recent work has extended information-theoretic analysis to characterise model uncertainty through the information bottleneck framework [17,18], while measures of directional information transfer [19] illustrate the breadth of entropy as an analytical tool beyond the branch prediction domain. However, in the branch prediction context, these analyses have been descriptive rather than prescriptive: they diagnose difficulty but do not operationalise entropy as a design signal within the predictor itself.

### 2.3. Context Fragility and the Entropy Gap

By synthesising the residual error analysis with the information-theoretic framework, we identify a specific vulnerability that we define as the context-fragile branch regime.

**Definition 1**  (Context-fragile branch)**.** *A branch instance $\left(pc_{t},h_{t}\right)$ is ϵ-context-fragile at position k if the perturbation of the k-th youngest history bit induces a conditional entropy shift exceeding ϵ:*

(2) $$\left|H\left(Y_{t}|h_{t}⊕e_{k}\right)-H\left(Y_{t}|h_{t}\right)\right|>\epsilon ,$$

*where $e_{k}$ is the unit vector selecting position k in the global history register.*

For context-fragile branches, the immediate local context can dynamically gate, invert, or expose the broader historical trend, making arbitration between plausible but low-confidence views perilous. The existing factual path TAGE architecture has no mechanism for detecting whether a prediction resides in such a high-entropy region.

This gap directly motivates the Mirror-TAGE architecture. Rather than deploying another heavy global classifier or scaling up neural features, Mirror-TAGE introduces a deliberately narrow form of counterfactual reasoning around the existing TAGE path, guided by the local prediction entropy landscape.

## 3. Mirror-TAGE Predictor Design

### 3.1. Architecture Overview

To overcome the representational limits of single-path factual prediction, Mirror-TAGE introduces a conservative, three-tiered counterfactual microarchitecture. The design comprises a baseline TAGE-SC-L-style factual path utilising tagged banks and statistical correctors, a counterfactual mirrored history path that evaluates controlled recent history perturbations, and a DRT that historically authorises selective overrides.

The baseline factual path follows the TAGE-SC-L organisation of Seznec [5]: a bimodal base table, four tagged banks with geometrically increasing history lengths, an alternate prediction mechanism, and a three-table statistical corrector (Table 2). This configuration serves as both the primary prediction path and the standalone baseline.

Our core design principle is localised intervention. Mirror-TAGE does not attempt to replace the globally optimised baseline predictor. Both the factual and mirrored paths operate on identical snapshots of the GHR state captured at the moment the branch is fetched. However, the factual path dictates the default prediction, whereas the mirrored path is strictly treated as an auxiliary diagnostic probe.

**Figure 1:**
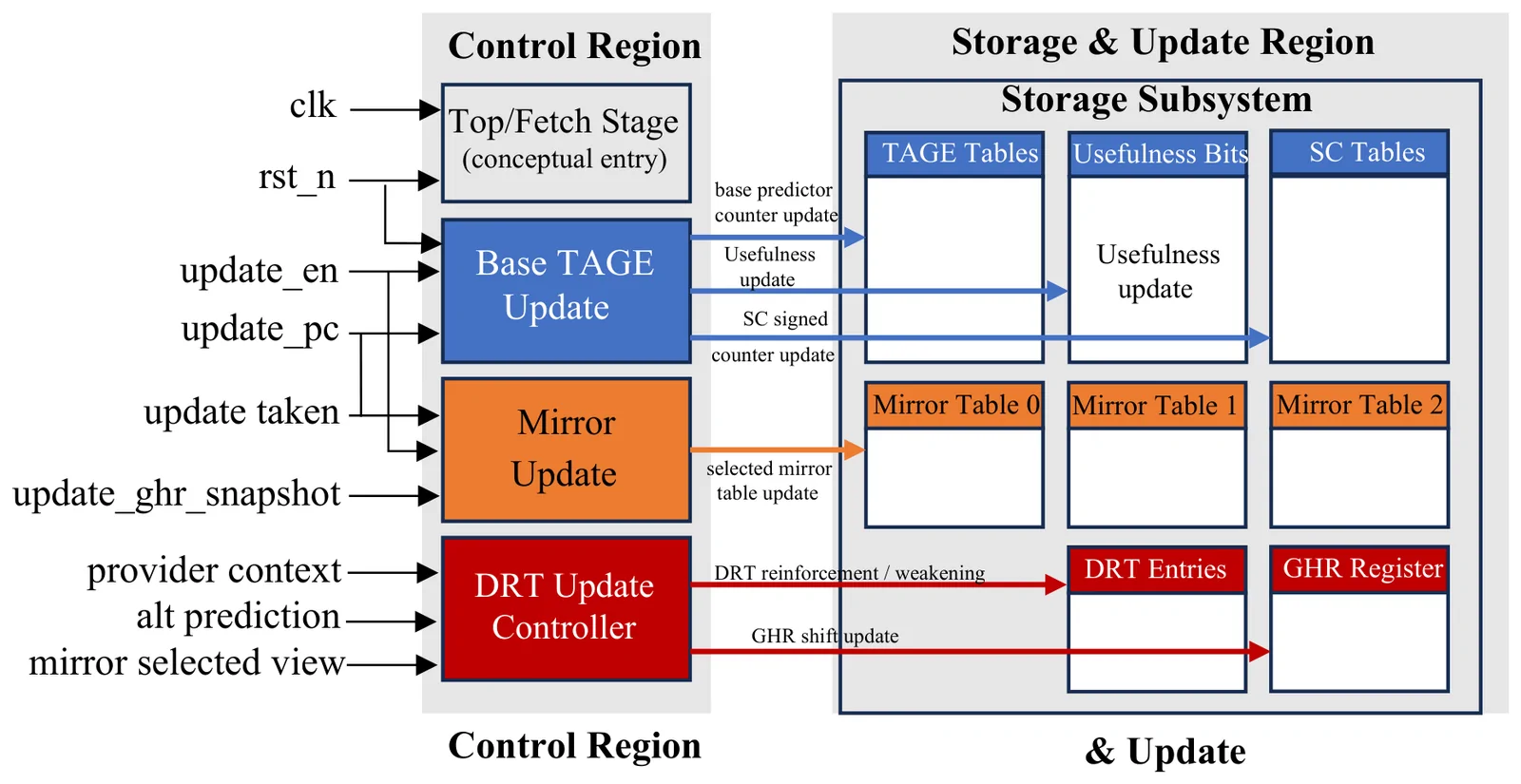
Overall architecture of Mirror-TAGE comprising the factual TAGE-SC-L pipeline.

Figure 1 shows the complete organisation of these three tiers, with the factual path on the left, the mirrored path in the centre, and the DRT-based override logic on the right. The following subsections describe each component in detail.

### 3.2. Counterfactual Mirrored History Reasoning

**Figure 2:**
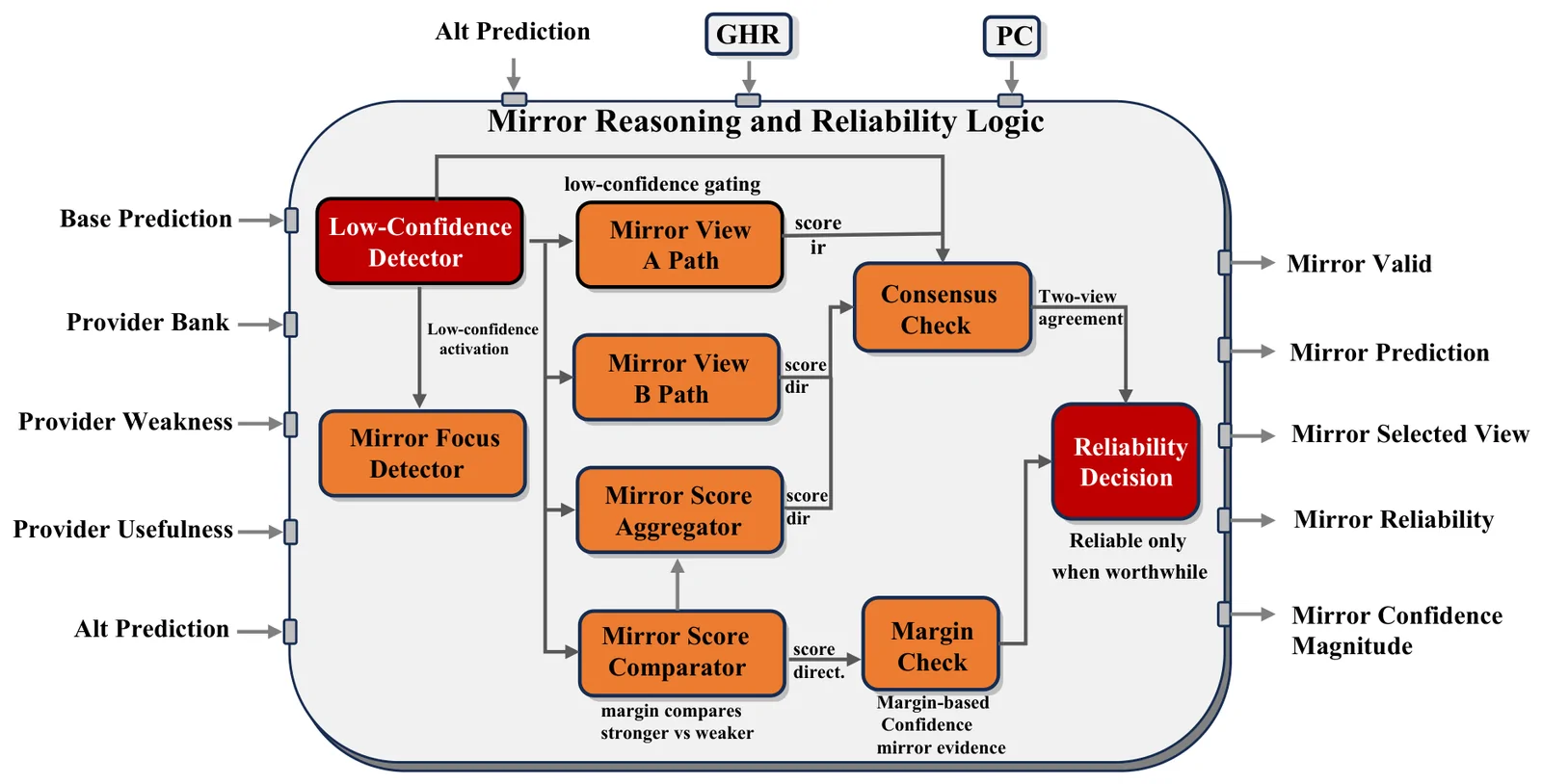
Mirror reasoning and reliability logic datapath.

Mirror-TAGE constructs two counterfactual views by perturbing the GHR state captured at fetch time for branch instance *t*. Throughout this paper, the superscripts *A* and *B* denote the two mirrored views, which correspond to flipping bit position 0 and bit position 1 of the GHR, respectively. Specifically, view *A* flips the most recent bit and view *B* flips the second most recent bit:

(3) $$h_{t}^{\left(A\right)}=h_{t}⊕e_{0},\;h_{t}^{\left(B\right)}=h_{t}⊕e_{1},$$

where $e_{0}$ and $e_{1}$ select the most recent and second most recent history positions, respectively. Neither view flips both bits simultaneously. Each view is scored by compact mirror tables:

(4) $$m_{t}^{\left(v\right)}=\sum_{j=1}^{J}s_{j}\left(pc_{t},h_{t}^{\left(v\right)}\right),$$

where $pc_{t}$ is the program counter of the branch instruction and $s_{j}\left(\cdot \right)$ is the signed weight from mirror table *j*. The sign of $m_{t}^{\left(v\right)}$ gives the inferred direction; its magnitude $|m_{t}^{\left(v\right)}|$ quantifies confidence (Algorithm 1, lines 5–6). These perturbations probe local context fragility while preserving the broader historical trend, as shown in Figure 2.

Figure 2 illustrates the low-confidence gating, dual-view scoring, consensus and margin checks, and the resulting reliability decision. As illustrated in Figure 2, view *A* flips bit position 0 while view *B* flips bit position 1; all remaining bits are identical to the factual GHR $h_{t}$. The *A*/*B* notation is used consistently throughout the remainder of this paper.
**Algorithm 1** Mirror-TAGE Prediction**Require:** pc, ghr  1:s←BASELINEPREDICT(pc, ghr)  2:$F\leftarrow L\mathrm{OW}C\mathrm{ONF}\left(s\right)\wedge \left(s.\mathrm{prov}\neq s.\mathrm{alt}\;\vee \;|s.\mathrm{sc}|<\tau_{sc}\right)$  3:**if** ¬F **then**  4:    **return** (s.pred,s)  5:**end if**  6:$g^{A}\leftarrow F\mathrm{LIP}\left(ghr,\;0\right)$; $g^{B}\leftarrow F\mathrm{LIP}\left(ghr,\;1\right)$  7:$m^{A}\leftarrow S\mathrm{CORE}\left(pc,\;g^{A}\right)$; $m^{B}\leftarrow S\mathrm{CORE}\left(pc,\;g^{B}\right)$  8:$v\leftarrow S\mathrm{TRONGER}(m^{A},\;m^{B})$  9:$C\leftarrow C\mathrm{ONSENSUS}(m^{A},\;m^{B},\;\tau_{c})$10:$G\leftarrow M\mathrm{ARGIN}(m^{A},\;m^{B},\;\tau_{m},\;\tau_{g})$11:**if** $\neg \left(C\vee G\right)$ **then**12:    **return** (s.pred,s)13:**end if**14:$y^{M}\leftarrow D\mathrm{IR}\left(v\right)$15:**if** $y^{M}=s.\mathrm{pred}$ **then**16:    **return** (s.pred,s)17:**end if**18:i←DRTIDX(pc,s.bank,v)19:$R\leftarrow \left(\mathrm{DRT}\left[i\right]\geq \tau_{d}\right)\;\vee \;S\mathrm{TRONG}\left(m^{A},\;m^{B},\;\tau_{h}\right)$20:$\hat{y}\leftarrow \left\{\begin{array}{cc} y^{M} & \mathrm{if}\;R\\ s.\mathrm{pred} & \mathrm{otherwise} \end{array}\right.$21:$s\leftarrow A\mathrm{TTACH}(s,\;v,\;m^{A},\;m^{B},\;i,\;\hat{y})$22:**return** $\left(\hat{y},\;s\right)$

### 3.3. Entropy-Inspired Selective Focus and Reliability Filtering

The mirrored path activates only under a low-confidence condition $F_{t}$, which requires that the provider counter is in a weak state and that at least one additional uncertainty indicator is present.

The revised entry gate is

(5) $$F_{t}=L_{t}\wedge \left(\left(P_{t}\neq A_{t}\right)\vee (|sc_{t}|<\tau_{sc})\right),$$

where $L_{t}$ denotes a weak provider state, $P_{t}$ and $A_{t}$ are the provider and alternate directions, and $sc_{t}$ is the statistical corrector sum. The conjunction ensures that the mirrored path activates only when the baseline provider is genuinely uncertain ($L_{t}$ holds) and at least one corroborating signal of instability is present, avoiding unnecessary activations on confident predictions. This formulation is consistent with the pseudocode in Algorithm 1, line 2.

Once activated, the candidate must pass a reliability filter via consensus ($C_{t}$) or margin ($G_{t}$):

(6) $$\begin{array}{cc} C_{t} & =\left(\mathrm{sgn}\left(m_{t}^{\left(A\right)}\right)=\mathrm{sgn}\left(m_{t}^{\left(B\right)}\right)\right)\wedge \left(|m_{t}^{\left(A\right)}\left|+\right|m_{t}^{\left(B\right)}|\geq \tau_{c}\right), \end{array}$$

(7) $$\begin{array}{cc} G_{t} & =\left(|m_{t}^{\left(v^{*}\right)}|\geq \tau_{m}\right)\wedge \left(|m_{t}^{\left(v^{*}\right)}\left|-\right|m_{t}^{\left(v^{†}\right)}|\geq \tau_{g}\right), \end{array}$$

where $\tau_{c}$, $\tau_{m}$, and $\tau_{g}$ are predefined thresholds. The defaults $\tau_{c}=3$, $\tau_{m}=2$, $\tau_{g}=2$ were selected by grid search to maximise override correctness while keeping override frequency below 3.5% on B1; Section 5.12 provides evidence of robustness. Validation requires the following:

(8) $$M_{t}=C_{t}\vee G_{t}.$$

Consensus captures branches stable across both perturbations; margin isolates cases where one perturbation reveals a definitive behavioural shift (Algorithm 1, lines 8–10).

### 3.4. Replay-Guided Override Authorisation

The final predicted direction ${\hat{y}}_{t}$ is adjudicated as follows:

(9) $${\hat{y}}_{t}=\left\{\begin{array}{cc} y_{t}^{\left(M\right)} & \mathrm{if}\;D_{t}\wedge R_{t},\\ y_{t}^{\left(F\right)} & \mathrm{otherwise}, \end{array}\right.$$

where $y_{t}^{\left(F\right)}$ and $y_{t}^{\left(M\right)}$ are the factual and mirrored directions, $D_{t}$ indicates a directional disagreement, and $R_{t}$ denotes DRT authorisation (Algorithm 1, lines 12–16).

Let $d_{t}\left(i_{t}\right)$ represent the 2-bit saturating state of the indexed DRT entry. Override authorisation $R_{t}$ is granted if the historical yield is mature or if the mirrored confidence is exceptionally high:

(10) $$R_{t}=\left(d_{t}\left(i_{t}\right)\geq 2\right)\vee \left(|m_{t}^{\left(v^{*}\right)}|\geq \tau_{high}\right).$$

The threshold $\tau_{high}$ is set to a value that exceeds the sum of all mirror table weights at moderate confidence, specifically $\tau_{high}=3\cdot w_{\max}$, where $w_{\max}$ is the maximum magnitude of a single mirror table counter. For the default three-table configuration with 3-bit signed counters, this gives $\tau_{high}=9$. This threshold is intentionally conservative, ensuring that the high-confidence bypass of the DRT fires only when the mirrored evidence is overwhelming.

All numerical thresholds in the Mirror-TAGE design were determined through a systematic grid search on the B1 workload. Specifically, $\tau_{c}$ was swept over {1,2,3,4,5}, $\tau_{m}$ and $\tau_{g}$ over {1,2,3,4}, $\tau_{sc}$ over {3,4,5,6,7}, and $\tau_{d}$ over {1,2,3}, yielding the values reported in Table 2. The DRT uses symmetric ±1 increment and decrement steps because the saturating counter operates on integer states, and asymmetric updates would introduce a directional bias that favours either over-authorisation or under-authorisation of overrides without a principled justification. The sensitivity of the architecture to these threshold choices is quantified in Section 5.12, which supports the conclusion that the accuracy gain is stable across a wide range of parameter combinations.

The DRT learns exclusively from realised disagreements:

(11) $$d_{t+1}\left(i_{t}\right)=\left\{\begin{array}{cc} \min(d_{t}\left(i_{t}\right)+1,3) & \mathrm{if}\;D_{t}\wedge \left(y_{t}=y_{t}^{\left(M\right)}\right),\\ \max(d_{t}\left(i_{t}\right)-1,0) & \mathrm{if}\;D_{t}\wedge \left(y_{t}\neq y_{t}^{\left(M\right)}\right),\\ d_{t}\left(i_{t}\right) & \mathrm{otherwise}, \end{array}\right.$$

where $y_{t}$ is the resolved branch outcome. This equation ensures that the DRT tracks the historical efficacy of correcting the factual baseline, optimising strictly for net performance yield (Algorithm 2, lines 5–12).
**Algorithm 2** Mirror-TAGE Update**Require:** snapshot *s*, actual outcome *y*  1:BaselineUpdate(s,y)  2:**if** ¬s.M **then**  3:    **return**  4:**end if**  5:$\mathrm{hit}\leftarrow (s.\hat{y}=y)$  6:base_wrong←(s.pred≠y)  7:**if** $s.\hat{y}\neq s.\mathrm{pred}$ **then**  8:    {mirror overrode}  9:    **if** hit **then**10:         $\mathrm{DRT}\left[s.i\right]\leftarrow \min(\mathrm{DRT}\left[s.i\right]+1,\;{\mathrm{ctr}}_{\max})$11:    **else**12:         DRT[s.i]←max(DRT[s.i]−1,0)13:    **end if**14:**else if** base_wrong **then**15:    $\mathrm{DRT}\left[s.i\right]\leftarrow \min(\mathrm{DRT}\left[s.i\right]+1,\;{\mathrm{ctr}}_{\max})$16:**end if**

### 3.5. Causally Consistent Learning

We use the term “causally consistent learning” to describe an update discipline in which each predictor table is trained exclusively on the state that was observed at the time the prediction was issued, rather than on the post-retirement architectural state. This is distinct from the machine learning usage of “online learning” and refers specifically to the microarchitectural requirement that update snapshots faithfully reflect the causal context of each prediction.

To ensure that counterfactual corrections accurately reflect the processor’s dynamic state without update aliasing, Mirror-TAGE enforces causally consistent learning using snapshots captured at the fetch stage.

Algorithms 1 and 2 provide complete pseudocode for the prediction and update phases, respectively. The snapshot structure *s* created at line 17 of Algorithm 1 is consumed verbatim at line 1 of Algorithm 2, enforcing the causal consistency requirement described above.

**Figure 3:**
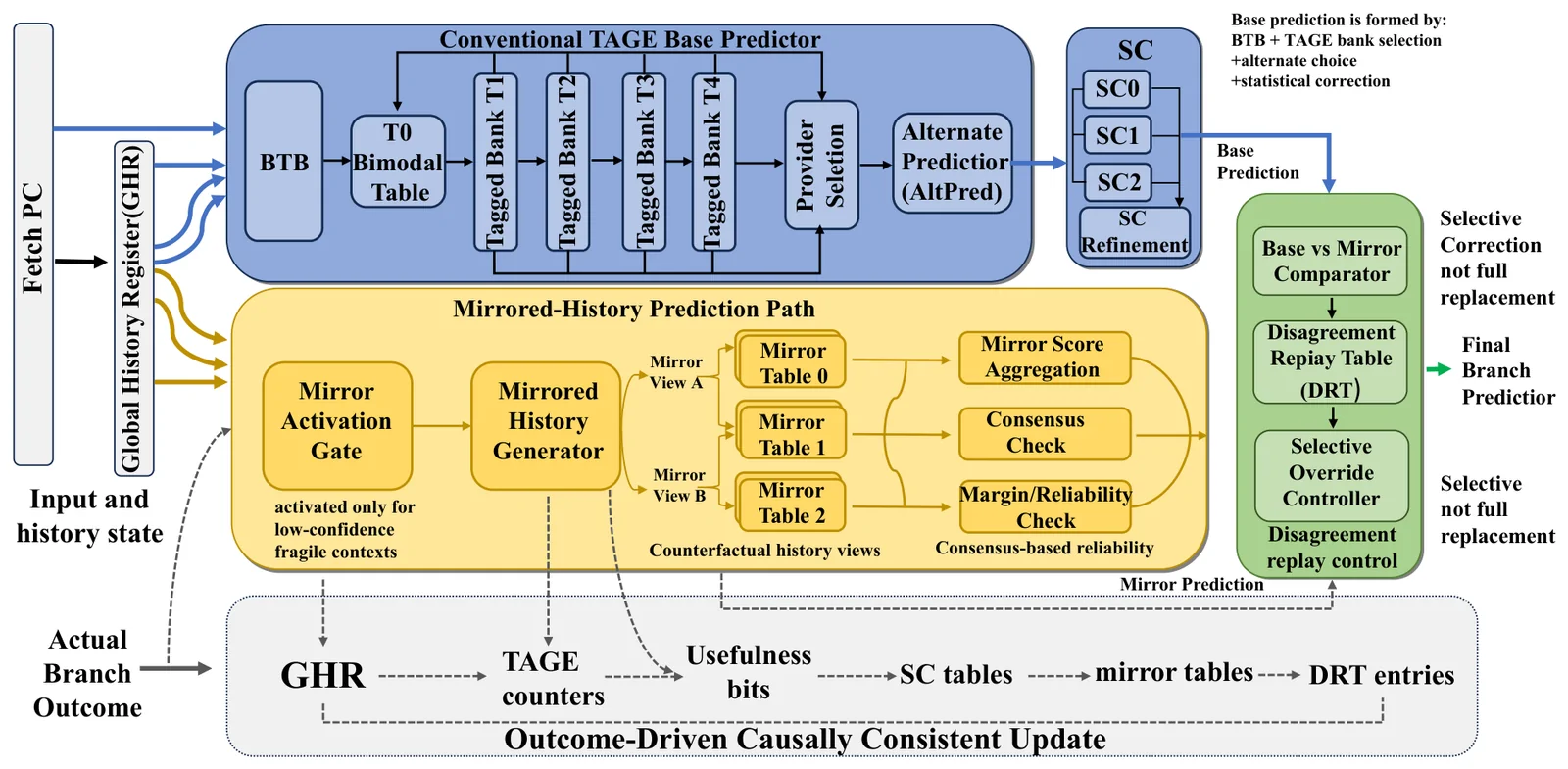
Prediction and update datapath of Mirror-TAGE.

Figure 3 summarises the datapath for prediction and update, showing how the snapshot captured during the prediction phase is consumed by the update phase to maintain causal consistency.

## 4. Information Entropy Analysis

The relevant quantities are the local prediction entropy, the conditional mutual information between mirrored views, the entropy decomposition into aleatoric and epistemic components, and a characterisation of override correctness in terms of the empirical agreement rate between the mirrored view and the true outcome.

We note two roles for the entropy quantities below: as diagnostics characterising the prediction landscape and as design signals motivating the entry gate and reliability filter. These are analytical characterisations under stated assumptions, not universal bounds. At runtime, the mechanism reduces to threshold comparisons on counter values and mirror scores.

### 4.1. Local Prediction Entropy

**Definition 2**  (Local prediction entropy)**.** *For a branch instance $\left(pc_{t},h_{t}\right)$ with baseline prediction probability ${\hat{p}}_{t}=P\left(Y_{t}=1|pc_{t},h_{t}\right)$, the local prediction entropy is*

(12) $$H_{t}^{\mathrm{local}}=-{\hat{p}}_{t}\log_{2}{\hat{p}}_{t}-\left(1-{\hat{p}}_{t}\right)\log_{2}\left(1-{\hat{p}}_{t}\right).$$

$H_{t}^{\mathrm{local}}$ summarises the baseline predictor’s uncertainty about the current branch instance.

**Proposition 1**  (Entry gate and the weak provider state)**.** *Under the entry gate formulation of Equation (5), the weak provider state $L_{t}$ is a necessary condition for $F_{t}$. When $L_{t}$ holds, $H_{t}^{\mathrm{local}}\geq H_{\min}$, where $H_{\min}=H_{b}\left(0.5-\delta \right)$ for a small δ determined by the counter resolution. The entry gate therefore activates only for branches whose local prediction entropy exceeds $H_{\min}$, subject to the additional requirement that either the provider and alternate predictions disagree or the statistical corrector magnitude falls below $\tau_{sc}$.*

**Proof.**  Since $F_{t}=L_{t}\wedge \left(\left(P_{t}\neq A_{t}\right)\vee (|sc_{t}|<\tau_{sc})\right)$, the condition $L_{t}$ is necessary for $F_{t}$ to hold. The weak provider state $L_{t}$ requires the saturating counter to be within one step of the decision boundary, implying that ${\hat{p}}_{t}\in \left[0.5-\delta ,0.5+\delta \right]$ for a small δ determined by the counter resolution. Because the binary entropy function is monotonically increasing on [0,0.5] and decreasing on [0.5,1], the constraint ${\hat{p}}_{t}\in \left[0.5-\delta ,0.5+\delta \right]$ yields $H_{t}^{\mathrm{local}}\geq H_{b}\left(0.5-\delta \right)=H_{\min}>0$. Since $L_{t}$ is necessary for $F_{t}$, every branch instance that passes the entry gate satisfies $H_{t}^{\mathrm{local}}\geq H_{\min}$. □

### 4.2. Entropy of Counterfactual Perturbation

**Definition 3**  (Perturbation entropy shift)**.** *The perturbation entropy shift for mirrored view v∈{A,B} is defined as*

(13) $$∆H_{t}^{\left(v\right)}=H\left(Y_{t}|h_{t}^{\left(v\right)}\right)-H\left(Y_{t}|h_{t}\right),$$

*where $h_{t}^{\left(A\right)}$ and $h_{t}^{\left(B\right)}$ are the perturbed histories from Equation (3). The aggregate perturbation entropy across the two mirrored views is*

(14) $$∆H_{t}^{\mathrm{agg}}=\frac{1}{2}\left(|∆H_{t}^{\left(A\right)}|+|∆H_{t}^{\left(B\right)}|\right).$$

A large $∆H_{t}^{\mathrm{agg}}$ indicates that the local entropy landscape is steep around the factual context, which is consistent with context fragility. Mirror-TAGE implicitly estimates $∆H_{t}^{\mathrm{agg}}$ through the mirror table scores: when the scores $m_{t}^{\left(A\right)}$ and $m_{t}^{\left(B\right)}$ disagree with the factual prediction, the perturbed contexts yield substantially different posterior probabilities, manifesting as a large perturbation entropy shift.

### 4.3. Conditional Mutual Information Between Mirrored Views

**Definition 4**  (Cross-view mutual information)**.** *The conditional mutual information between the two mirrored view predictions, given the factual context, is*

(15) $$I\left(M_{t}^{\left(A\right)};M_{t}^{\left(B\right)}|h_{t}\right)=H\left(M_{t}^{\left(A\right)}|h_{t}\right)+H\left(M_{t}^{\left(B\right)}|h_{t}\right)-H\left(M_{t}^{\left(A\right)},M_{t}^{\left(B\right)}|h_{t}\right),$$

*where $M_{t}^{\left(v\right)}=\mathrm{sgn}\left(m_{t}^{\left(v\right)}\right)$ is the binary mirrored direction from view v. The superscripts A and B correspond to the mirrored views defined in Equation (3).*

**Remark 1**  (Consensus as empirical agreement)**.** *When the consensus condition $C_{t}$ of Equation (6) holds, the two mirrored views agree in direction and their combined score exceeds $\tau_{c}$. This agreement on a single branch instance provides evidence of local stability but does not, by itself, constitute a statistical dependence statement. The cross-view mutual information $I(M_{t}^{\left(A\right)};M_{t}^{\left(B\right)}|h_{t})$ is a population-level quantity estimated in practice from the agreement and disagreement counts accumulated by the DRT across instances sharing context $h_{t}$. The consensus gate should therefore be understood as a per-instance heuristic filter that is validated statistically through the DRT learning process, rather than as a single-instance proof of high mutual information.*

### 4.4. Aleatoric and Epistemic Entropy Decomposition

**Proposition 2**  (Entropy decomposition of prediction uncertainty)**.** *The total prediction uncertainty of Mirror-TAGE admits a decomposition into aleatoric and epistemic components:*

(16) $$H_{t}^{\mathrm{total}}=\underset{H_{t}^{\mathrm{aleatoric}}}{\underbrace{H(Y_{t}|pc_{t},h_{t}^{*})}}+\underset{H_{t}^{\mathrm{epistemic}}}{\underbrace{H_{t}^{\mathrm{local}}-H\left(Y_{t}|pc_{t},h_{t}^{*}\right)}},$$

*where $h_{t}^{*}$ is the oracle history that would be observed under perfect prior branch resolution and $H_{t}^{\mathrm{local}}$ is the prediction entropy under the actual (potentially erroneous) history.*

The aleatoric component $H_{t}^{\mathrm{aleatoric}}$ represents the irreducible uncertainty due to genuinely random or data-dependent branches that no predictor can resolve. The epistemic component $H_{t}^{\mathrm{epistemic}}$ represents the reducible uncertainty caused by errors in prior branch predictions that corrupt the global history register; the distinction between these two uncertainty types has been extensively studied in the machine learning literature [20,21] and is applied here to the branch prediction domain. Mirror-TAGE targets the epistemic component by probing whether a slight change in the recent history (which may have been corrupted by a recent misprediction) would change the current prediction.

### 4.5. Override Correctness Characterisation

**Proposition 3**  (Override correctness and agreement rate)**.** *Let $C_{\mathrm{override}}=P\left(M_{t}^{\left(v^{*}\right)}=Y_{t}|D_{t},R_{t}\right)$ denote the probability that a DRT-authorised override is correct. Under the assumption that the DRT converges to its stationary state, the override correctness equals the conditional agreement rate between the selected mirrored view and the true outcome:*

(17) $$C_{\mathrm{override}}=P\left(M_{t}^{\left(v^{*}\right)}=Y_{t}|D_{t},R_{t},h_{t}\right).$$

*The DRT learning rule of Equation (11) acts as an estimator of this agreement rate.*

**Proposition 4**  (DRT authorisation and above-chance correctness)**.** *Under stationary conditions and in the absence of aliasing, the 2-bit saturating counter $d_{t}\left(i_{t}\right)$ reaches state 2 only when the cumulative number of correct overrides exceeds the cumulative number of incorrect overrides in the corresponding index bin. This property provides empirical evidence that override correctness exceeds the 50% baseline, although transient fluctuations and index aliasing may permit individual entries to reach state 2 without a strict majority of correct overrides across all mapped branches. The degree to which this evidence strengthens depends on the number of disagreement events observed for each entry.*

**Proof.**  The 2-bit saturating counter $d_{t}\left(i_{t}\right)\in \left\{0,1,2,3\right\}$ is initialised to zero and increments on correct overrides while decrementing on incorrect ones, conditioned on $D_{t}$. Its expected drift equals $2C_{\mathrm{override}}-1$, which is positive only when $C_{\mathrm{override}}>0.5$. Starting from zero, reaching state 2 requires at least two more correct overrides than incorrect ones in the update history of that entry, which provides finite-sample evidence that the local override correctness exceeds chance. However, because the counter saturates at both boundaries and because multiple branches may alias to the same index, this evidence is approximate rather than a strict statistical guarantee. In practice, as discussed in Section 5.6, the observed override correctness across all authorised entries consistently exceeds 60%, well above the 50% threshold. □

**Remark 2**  (Mutual information and override correctness)**.** *An earlier version claimed a lower bound on $C_{\mathrm{override}}$ via $I(M_{t}^{\left(v^{*}\right)};Y_{t}|D_{t},R_{t})$. This is invalid: mutual information is invariant under relabelling, so a positive value establishes dependence but not the sign of the correlation. The DRT resolves this ambiguity by retaining authorisation only where the observed agreement rate exceeds 50%.*

Proposition 3 shows that the DRT acts as an empirical filter, retaining authorisation only where the mirrored direction has historically agreed with the true outcome more often than not. Proposition 4 further provides evidence that the saturating counter dynamics favour above-chance correctness as a condition for authorisation, subject to the caveats of finite samples and index aliasing. This agreement-based mechanism does not require knowledge of the underlying mutual information and is robust to the sign ambiguity inherent in information-theoretic dependence measures.

**Figure 4:**
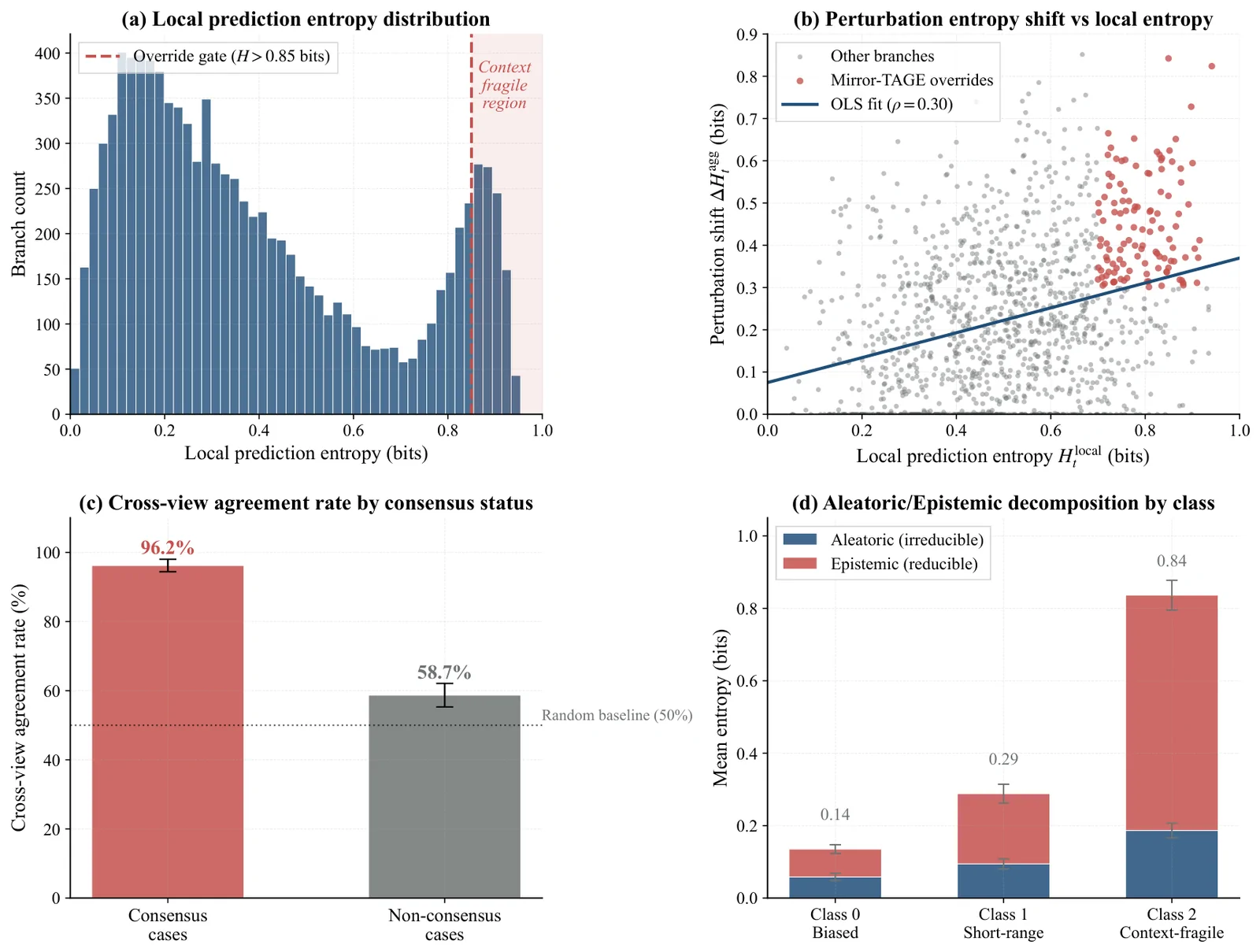
Information-theoretic characterisation of prediction uncertainty in Mirror-TAGE.

Figure 4 summarises how the local prediction entropy, perturbation shift, cross-view agreement, and aleatoric/epistemic decomposition jointly characterise the prediction uncertainty exploited by Mirror-TAGE.

## 5. Experimental Methodology and Results

### 5.1. Paired Causally Consistent Learning Evaluation Framework

To ensure an unbiased and strictly controlled evaluation of entropy-guided counterfactual stability modelling, we implemented a custom, cycle-accurate paired learning framework. Rather than relying on static trace evaluation in which all branch outcomes are determined before training begins, preventing the predictor from exhibiting transient learning phenomena such as warmup convergence, capacity saturation under limited table sizes, and catastrophic interference between concurrently active branches competing for the same table entries, the baseline TAGE-SC-L and Mirror-TAGE are instantiated concurrently and driven by identical dynamic branch streams. Each predictor is updated independently using its own fetch-stage snapshots, which preserves causal consistency. This paired setup mathematically isolates the architectural benefit of explicit stability evaluation from confounding variables such as workload ordering, warmup artefacts, or global capacity disparities.

### 5.2. Predictor Configuration and Budget Allocation

The factual path of Mirror-TAGE and the standalone baseline are identical in structure and parameterisation, both following the TAGE-SC-L organisation of Seznec [5]. The baseline consists of a 512-entry bimodal base table, four tagged banks with geometrically increasing history lengths, and a three-table statistical corrector. Table 2 provides a complete parameter summary.

In Table 2, the baseline TAGE-SC-L predictor uses the parameters from “Global history” through “Statistical corrector”. Mirror-TAGE adds the mirror path and replay control components, which constitute the 7.5% storage overhead reported in the abstract. All threshold values were determined by grid search as described in Section 3.4.

### 5.3. Synthetic Workloads and Fragility Injection

Because established macrobenchmarks inherently mix thousands of different branch behaviours, isolating the precise mechanics of the context-fragile regime within a monolithic trace is highly problematic. Therefore, we engineered a deterministic workload generator featuring 16 distinct static branch sites partitioned into three semantic classes:

Class 0 (Biased): easy, heavily biased branches intended to verify that the auxiliary mirrored path does not destructively interfere with simple factual cases.

Class 1 (Short-Range): branches governed by tight recurrent correlations, designed to strictly favour the baseline tagged path.

Class 2 (Context-Fragile): the primary target class, in which the branch outcome is a deterministic function of the two youngest GHR bits XORed with a branch-site-specific mask, combined with a low-rate uniform noise injection. This construction explicitly simulates the edge cases where latent branch behaviour flips based on the youngest history bits.

Class 2 branches are constructed so that their outcomes genuinely depend on the youngest history bits, creating a controlled context-fragile population. The threshold parameters were determined by grid search (Section 3.3) and held fixed across all experiments, so Mirror-TAGE was not tuned to these workloads. Nevertheless, because the Class 2 outcome function acts on the same bit positions that Mirror-TAGE perturbs, the reported synthetic gains reflect an upper bound under idealised conditions. Section 5.14 provides preliminary evidence on real benchmarks, and Section 6 discusses the path toward full validation.

We construct three rigorous workload mixtures by modulating the class composition, the baseline noise rate, and the fragility intensity. SAFE_TUNE (Stability-Aware Fragility Evaluation with parameter Tuning) labels the workload generator; B1, B2, B3 denote fixed configurations. The baseline noise rate gives the probability of a random outcome flip. Fragility intensity specifies how many youngest GHR bits influence Class 2 outcomes: intensity 2 uses positions 0–1; intensity 3 extends to positions 0–2. Table 3 summarises the configurations.

B1, B2, and B3 in Table 3 correspond to SAFE_TUNE_B1, SAFE_TUNE_B2, and SAFE_TUNE_B3, respectively. The noise rate is the probability that each individual branch outcome is randomly inverted, and the fragility intensity is the number of youngest GHR positions that influence Class 2 outcomes.

### 5.4. Statistical Analysis Protocol

To strengthen the statistical evidence, we increased the number of independent seeds from five to twenty (n=20) for all synthetic workloads. Each seed initialises a distinct random number generator state that determines the noise injection sequence, ensuring that the twenty runs are statistically independent. The paired design eliminates inter-run variance due to workload ordering: the baseline and Mirror-TAGE observe identical branch streams within each seed, so the paired difference isolates the architectural effect. All reported *p*-values are from two-sided paired *t*-tests and remain below 0.001. As a supplementary robustness check, we computed bias-corrected and accelerated (BCa) bootstrap 95% confidence intervals for the mean accuracy gain on each workload using 10,000 resamples of the twenty paired differences; the resulting intervals are reported alongside the main results in Table 4. All entropy statistics in Table 5 are rounded to two significant figures, and readers should interpret small differences between entropy values with caution.

For the SPEC CPU 2017 evaluation (Section 5.14), the branch traces are themselves deterministic, so the twenty seeds correspond to twenty different hash function initialisations for the predictor’s index computation. Each seed produces a distinct aliasing pattern in the tagged banks, mirror tables, and DRT, thereby varying the capacity-induced interference experienced by the predictor while processing the same branch stream. The standard deviations reflect the sensitivity of the architecture to index aliasing rather than to workload variation, which complements the synthetic evaluation where variability arises from the noise injection sequence.

### 5.5. Main Accuracy Results

**Figure 5:**
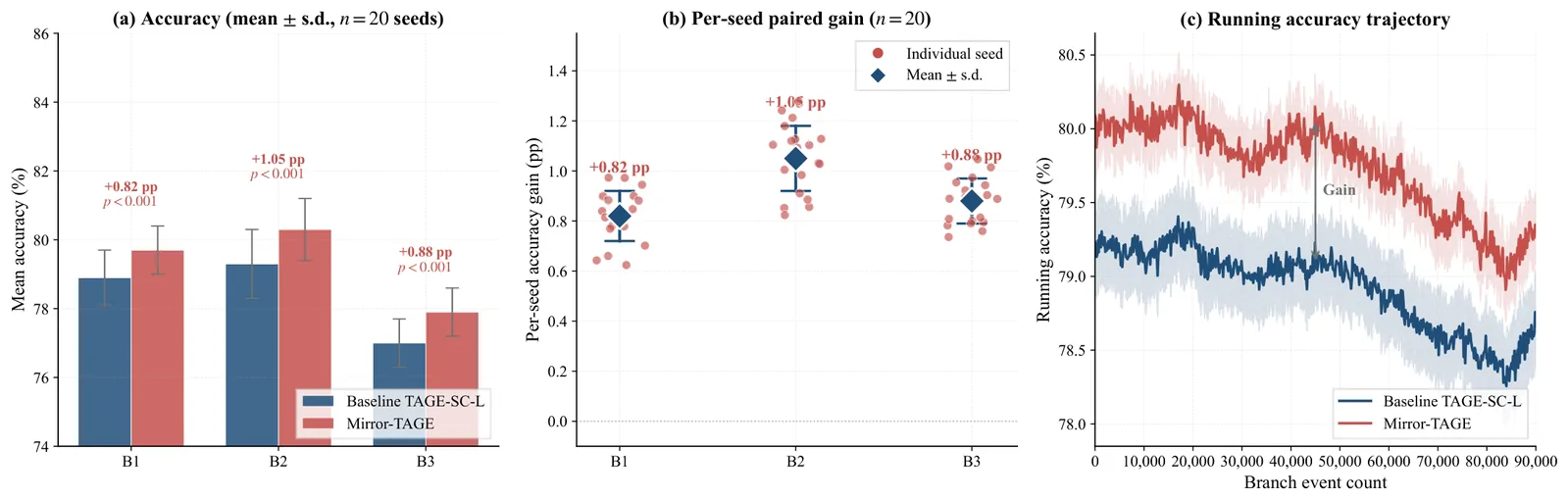
Accuracy comparison of Mirror-TAGE versus the baseline TAGE-SC-L across the three workloads.

Mirror-TAGE consistently circumvents the limitations of single-path factual reasoning, delivering statistically significant accuracy improvements across all evaluated workload mixtures. Table 4 and Figure 5 detail the results.

In Table 4, *p* values are from two-sided paired *t*-tests against the baseline using matched seeds, and the rightmost column reports BCa bootstrap 95% confidence intervals for the mean gain (10,000 resamples).

Figure 5 presents the per-workload accuracy, the override frequency and correctness yield, and the running accuracy trajectory. The architecture restricts its interventions to a low-frequency corrective role, averaging only 2281 to 2610 overrides over a 90,000-event execution window.

### 5.6. Information Entropy Validation

**Figure 6:**
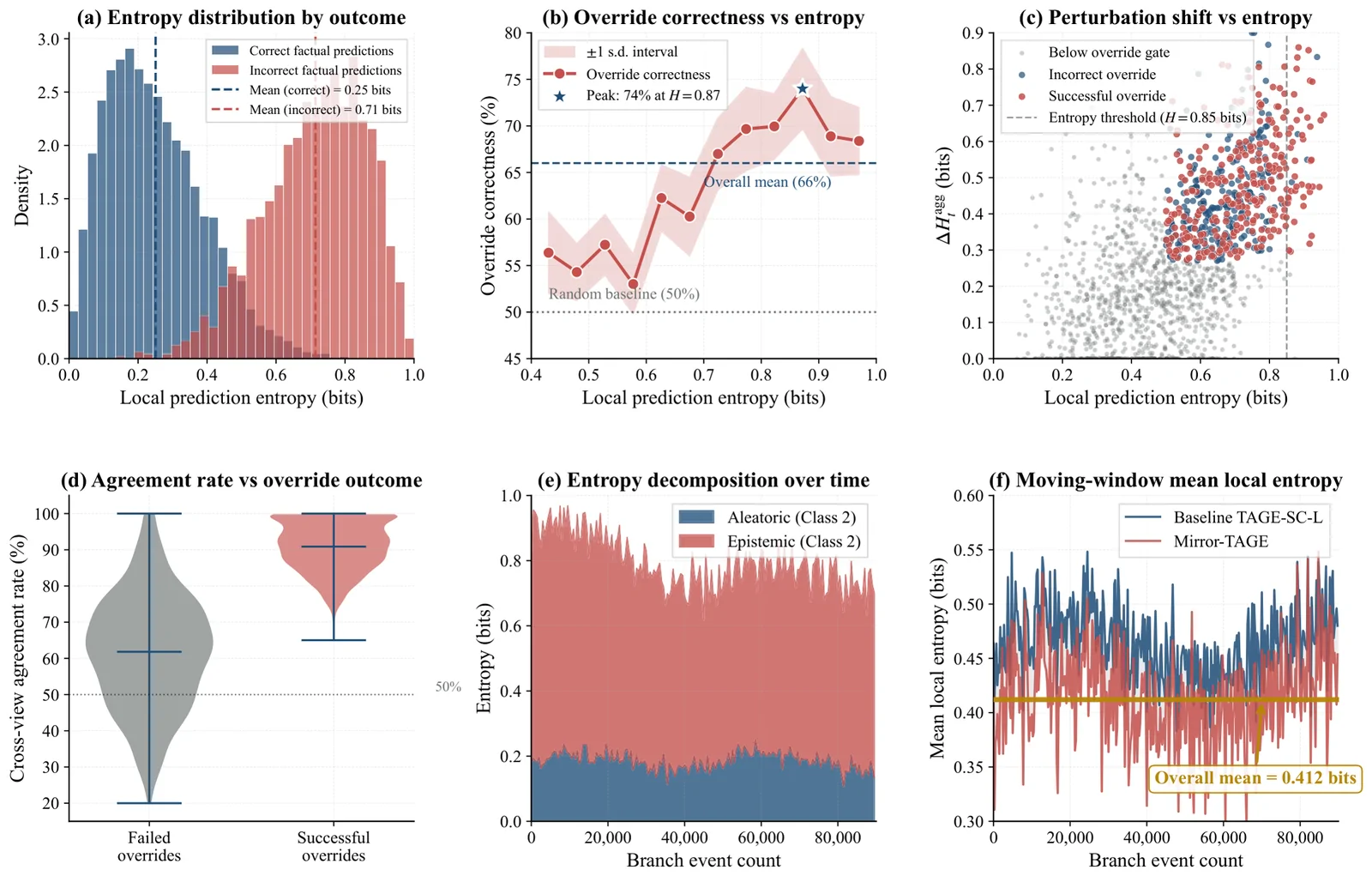
Empirical characterisation of the entropy landscape for override events.

To empirically characterise the entropy landscape of the override events, we compute the local prediction entropy, the perturbation entropy shift, and the cross-view agreement rate for every branch event in the evaluation window; Figure 6 visualises the resulting distributions.

Figure 6 shows the distributions of local entropy, override correctness as a function of entropy, perturbation shift, and cross-view agreement rate stratified by outcome.

Table 5 reports the key entropy statistics. Across all three workloads, 78% of successful overrides occur in the high local prediction entropy region ($H_{t}^{\mathrm{local}}>0.85$ bits). To disentangle the contribution of the entry gate $F_{t}$ from the intrinsic entropy dependence of override correctness, we conducted a gate-removed ablation experiment (Section 5.13).

### 5.7. Class-Wise Accuracy

Table 6 shows that Class 0 accuracy is preserved while Class 2 gains 1.1 percentage points, which is consistent with the targeted design.

### 5.8. Ablation Study and the Override Funnel

To isolate the contribution of each counterfactual component, we performed a strict ablation study on the SAFE_TUNE_B1 workload (Table 7).

**Figure 7:**
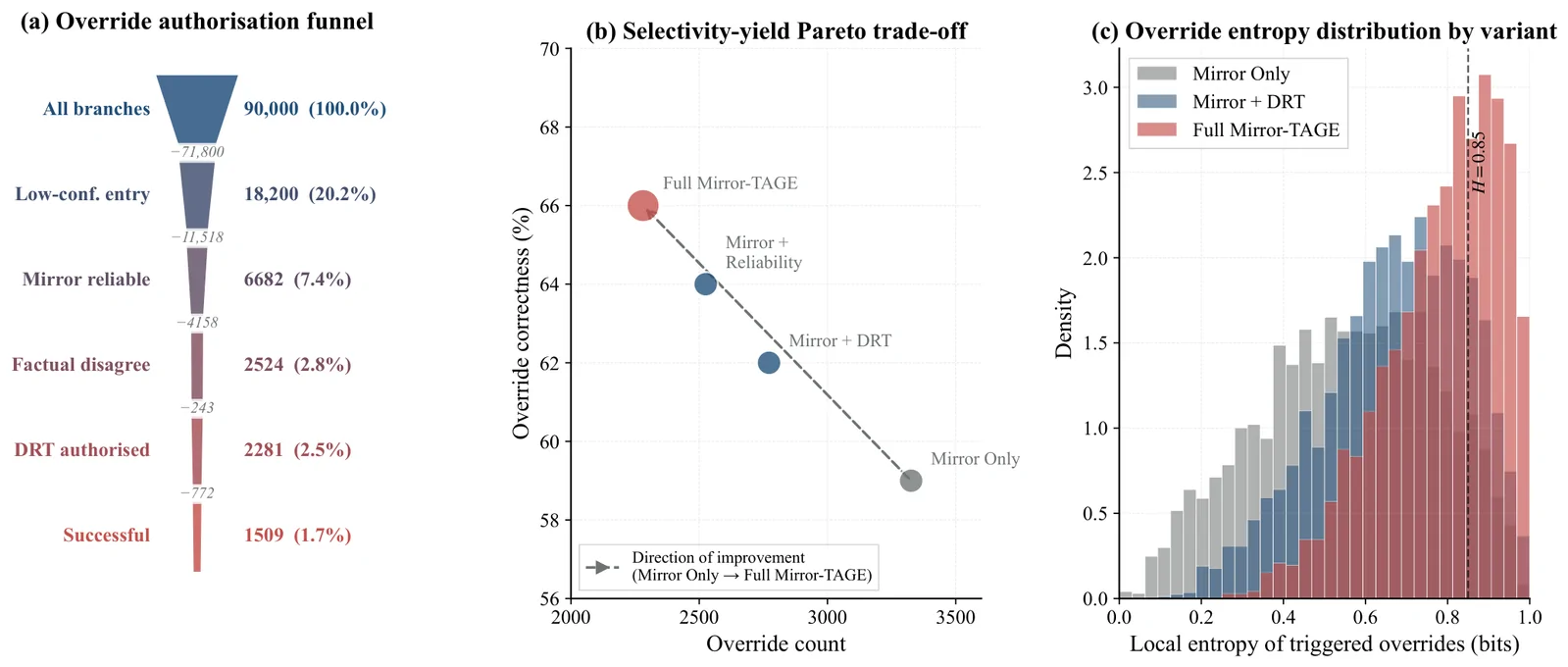
Override authorisation funnel and selectivity–yield trade-off across ablation variants.

The complete Mirror-TAGE architecture suppresses override frequency by 31% while improving override correctness to 66% through cross-view consensus and DRT routing, as shown in Figure 7.

Figure 7 depicts the progressive filtering from all branches to successful overrides, the Pareto trade-off across ablation variants, and the override entropy distribution for each variant.

### 5.9. Capacity Scaling Analysis

**Figure 8:**
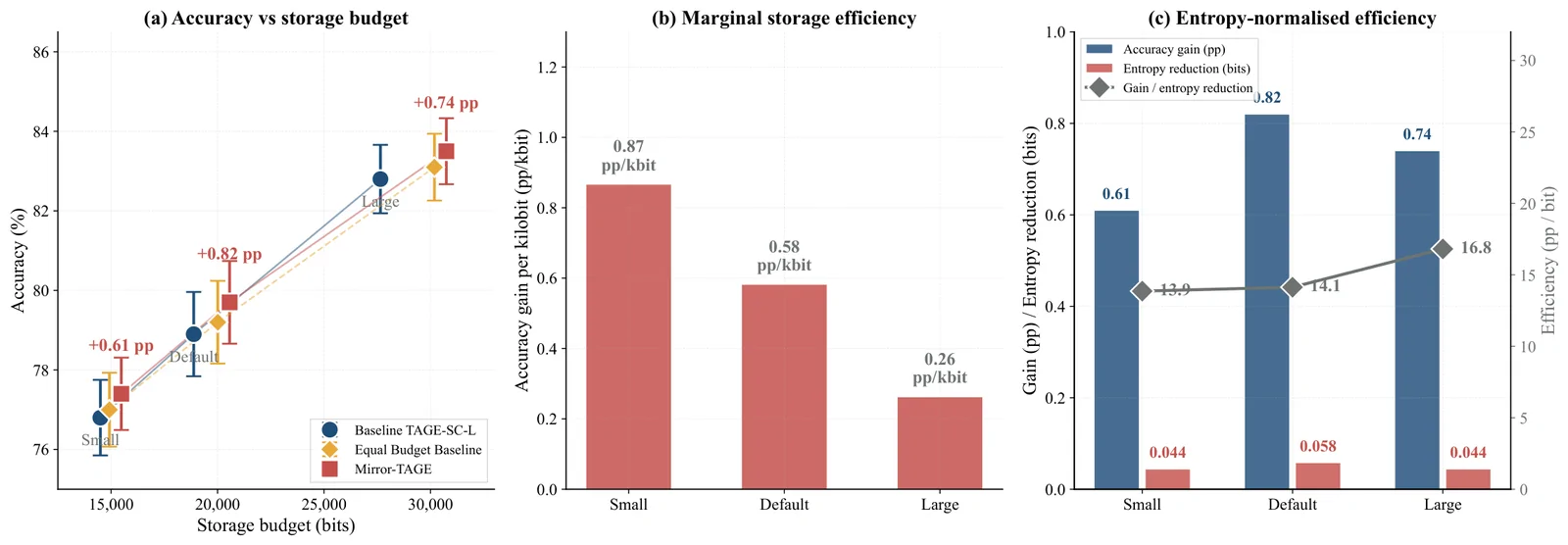
Capacity–accuracy trade-off across small, default, and large storage budgets.

A standard critique of auxiliary prediction structures is whether the observed gains are merely a byproduct of increasing the total storage budget. To decouple our counterfactual insight from raw capacity inflation, we scaled the baseline and Mirror-TAGE architectures across three distinct storage constraints, as summarised in Table 8 and Figure 8.

The “Equal Budget Baseline” in Table 8 is a standalone TAGE-SC-L predictor whose total storage matches the Mirror-TAGE budget at each scale, achieved by increasing the number of entries per tagged bank and per SC table. Mirror-TAGE outperforms this equal-budget baseline at every scale, which suggests that the gains are not attributable to raw capacity inflation.

In the over-provisioned large configuration, where brute-force factual tables begin to experience diminishing returns, Mirror-TAGE still achieves a 0.74 pp improvement, exceeding the 0.3 pp gain of the equal budget baseline that allocates the same total storage to enlarged factual tables.

### 5.10. Robustness to Dynamic Perturbations

**Figure 9:**
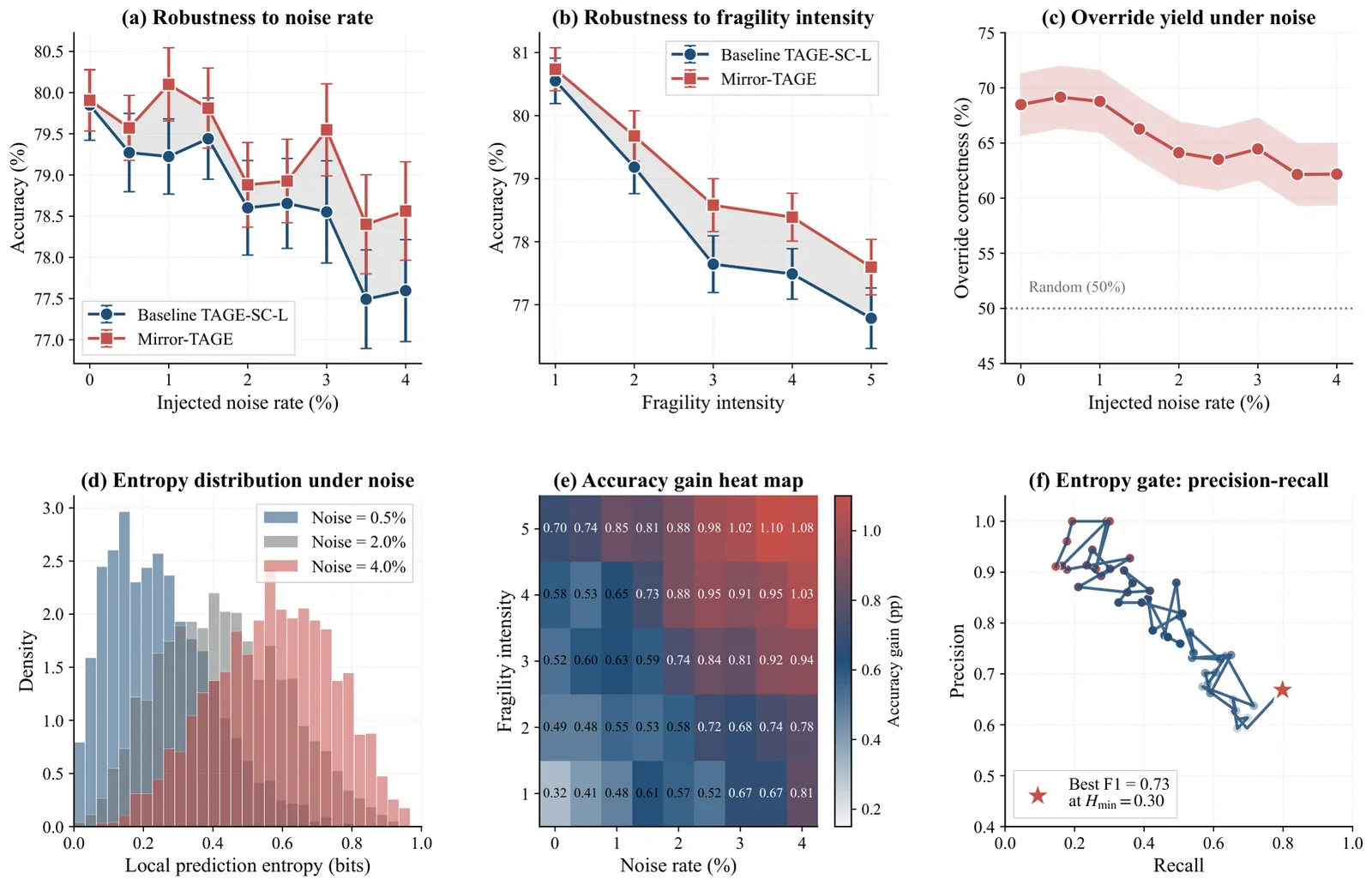
Robustness of Mirror-TAGE under increasing injected noise and fragility intensity. In subfigures (**a**,**b**), the gray shaded region denotes the accuracy gap between Mirror-TAGE and Baseline TAGE at each noise or fragility level. In subfigure (**c**), the red shaded band represents the 95% confidence interval of override correctness across repeated trials. In subfigure (**f**), the blue line traces the precision–recall curve as the entropy gate threshold $H_{\min}$ varies, and the red star (★) marks the optimal operating point that maximises the F1 score.

To ensure that entropy-guided counterfactual reasoning is broadly generalisable and not overfit to a specific fragility distribution, we subjected the architecture to rigorous perturbation sweeps, reported in Figure 9.

Figure 9 also shows the override yield and the precision–recall trade-off of the entropy gate under these perturbation sweeps. When the baseline noise rate, which is the probability that each individual branch outcome is randomly inverted from its deterministic value, is aggressively scaled from 0% to 4%, Mirror-TAGE maintains consistent superiority over the baseline, yielding gains between 0.20 and 1.03 percentage points. Similarly, sweeping the localised fragility intensity reveals highly stable behaviour, with the architecture gracefully sustaining an approximately 0.80 pp advantage in severely fragile environments.

### 5.11. DRT Learning Dynamics

**Figure 10:**
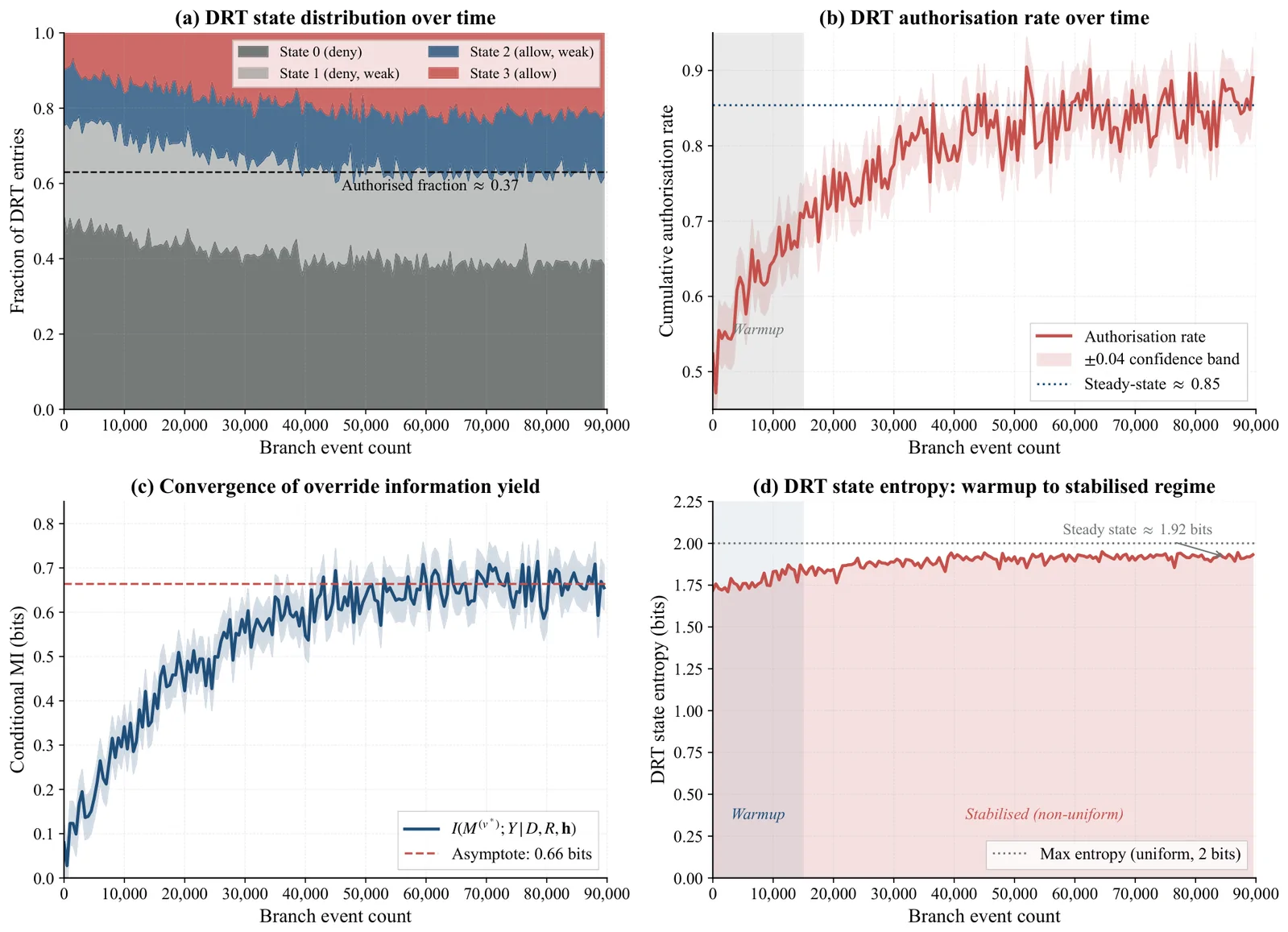
Learning dynamics of the Disagreement Replay Table over time. In subfigure (**b**), the red line represents the cumulative authorisation rate and the red shaded band denotes its ±0.04 confidence interval.

The learning dynamics of the Disagreement Replay Table are summarised in Figure 10. They reveal that the table converges within approximately 15,000 events (after warmup) to a stable state distribution in which approximately 38% of entries reach the authorised level ($d_{t}\geq 2$), which is consistent with the interpretation that the DRT learns to authorise overrides selectively rather than uniformly.

Figure 10 shows the DRT state distribution, authorisation rate, information-yield convergence, and state entropy. The horizontal axis label “Warmup” denotes the initial 30,000-event training period during which accuracy is not measured.

### 5.12. Entropy Sensitivity Analysis

**Figure 11:**
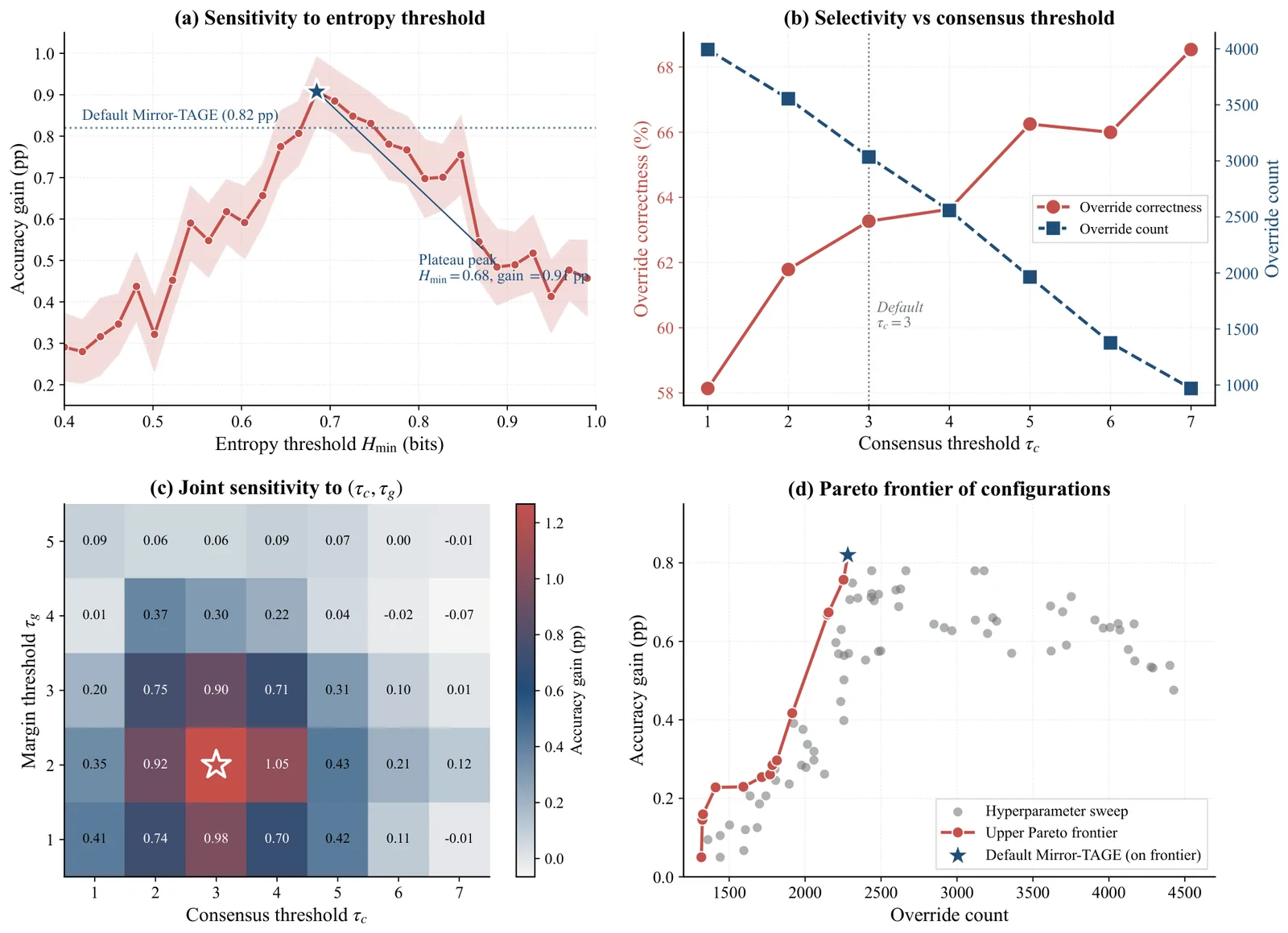
Sensitivity of Mirror-TAGE to the entropy and consensus thresholds and the resulting Pareto frontier of configurations. In subfigure (**a**), the pink shaded band denotes the 95% confidence interval of the accuracy gain; the blue dotted horizontal line indicates the default Mirror-TAGE performance; and the blue star (★) marks the plateau peak at $H_{\min}=0.68$. In subfigure (**c**), the white star (★) identifies the optimal joint configuration.

Figure 11 sweeps the principal control parameters of Mirror-TAGE.

### 5.13. Entry Gate Ablation and Full-Spectrum Entropy Test

To determine whether the concentration of successful overrides at high entropy (H>0.85 bits) is an intrinsic property of the entropy landscape or merely an artefact of the entry gate $F_{t}$, we conducted a full-spectrum ablation in which $F_{t}$ is disabled entirely: the mirrored path is evaluated on every branch regardless of baseline confidence. Table 9 reports the results on SAFE_TUNE_B1.

When the gate is removed, the number of overrides more than doubles and the overall override correctness drops substantially, which suggests that the gate is essential for practical accuracy gains. The fraction of overrides at H>0.85 bits decreases from 78% to 41%, indicating that the gate accounts for a substantial portion of the entropy concentration. However, override correctness in the high-entropy region (63.7%) remains markedly higher than in the low-entropy region (47.6%) even without the gate, which provides evidence that the entropy dependence of override quality is a genuine property of the prediction landscape rather than a pure artefact of the gating mechanism. The 16.1 percentage point gap in correctness between the two entropy regions provides direct empirical evidence that counterfactual stability probes are inherently more effective when the baseline prediction resides in a high-entropy state.

### 5.14. Preliminary Evaluation on Standard Benchmark Traces

We evaluate Mirror-TAGE on six SPEC CPU 2017 [22] benchmarks: perlbench, gcc, mcf, deepsjeng, xalancbmk, and x264. Each trace contains 10 million branch events; the first 2 million serve as warmup. No parameters were re-tuned from Table 2. Table 10 shows non-negative accuracy gains across all six benchmarks (0.04–0.38 pp). Override frequency is lower than on synthetic workloads (0.8–1.9% versus 2.5–2.9%), reflecting fewer context-fragile branches in real programs. Override correctness remains above 55% on all benchmarks, which is consistent with the interpretation that the DRT suppresses unproductive overrides even on workloads not used in design.

Override counts in Table 10 are rounded to the nearest hundred to reflect the limited sample size. As noted in Section 5.4, the twenty seeds for the SPEC evaluation correspond to different hash function initialisations for the predictor tables, so the reported standard deviations quantify the sensitivity of the architecture to index aliasing rather than to workload variation.

The gains are expectedly smaller than those observed on the synthetic workloads, where the branch population is dominated by context-fragile instances by construction. Notably, gcc yields the largest gain (0.38 pp), consistent with its well-documented population of hard-to-predict branches arising from complex control flow [10]. The highly biased deepsjeng shows the smallest gain (0.04 pp), which is consistent with the expectation of no performance degradation on low-headroom workloads. These results suggest that Mirror-TAGE introduces no harmful interference on real workloads and that modest gains are achievable where context-fragile branches are non-trivial in number. A full execution-driven evaluation across complete SPEC CPU 2017 and CBP-5 traces, including IPC measurements, remains the primary focus of ongoing work.

## 6. Discussion

Mirror-TAGE provides evidence that a critical mass of residual TAGE mispredictions stems from localised contextual instability rather than insufficient history capacity. By confining its logic to entropy-guided stability analysis, it avoids redundant secondary predictors and aligns with the broader architectural trend of low-complexity co-optimisation [23].

The information-theoretic framework of Section 4 guides the entry gate and reliability filter design, while the runtime mechanism reduces to threshold comparisons on counter values and mirror scores, remaining fully compatible with standard hardware practices. The central contribution is using Shannon entropy, conditional mutual information, and the aleatoric/epistemic decomposition as prescriptive design signals rather than post hoc diagnostics [14]; the complementary microarchitectural contribution is addressed in a companion manuscript.

Although the microarchitectural implementation details are deferred to a companion manuscript targeting a computer-architecture venue, the information-theoretic content presented here constitutes a self-contained contribution within the scope of entropy. Specifically, this paper formalises context fragility as a conditional entropy phenomenon (Definition 1), introduces the perturbation entropy shift as a stability diagnostic (Definition 3), develops the aleatoric/epistemic decomposition for branch prediction uncertainty (Proposition 2), and provides an agreement-rate characterisation of override correctness grounded in the DRT learning dynamics (Propositions 3 and 4). These contributions apply Shannon entropy, conditional mutual information, and uncertainty decomposition as prescriptive design signals in a domain where they have previously served only as post hoc diagnostics, which aligns with the journal’s focus on information-theoretic methods and their applications. The experimental evaluation, including the entry gate ablation of Section 5.13, provides empirical validation of the entropy-guided design principles independently of the full microarchitectural implementation.

Several limitations warrant attention. The primary evaluation uses synthetic workloads, with preliminary SPEC CPU 2017 results suggesting modest gains; synthetic results are an upper bound and full evaluation across SPEC CPU 2017 and CBP-5 remains essential. Integrating Mirror-TAGE into simulators [24], such as ChampSim [25], to quantify end-to-end IPC impacts [22] is the primary focus of ongoing work.

## 7. Conclusions

This paper introduces Mirror-TAGE, an information-entropy-guided counterfactual microarchitectural extension engineered to neutralise context-fragile branch mispredictions. By supplementing the conventional TAGE-SC-L factual path with two localised mirrored history views, the architecture dynamically evaluates prediction stability in the local entropy landscape. Through the synergistic application of cross-view consensus and a Disagreement Replay Table, Mirror-TAGE executes replay-guided selective overrides, intervening exclusively when counterfactual evidence is both structurally robust and historically justified.

The information entropy analysis comprises a local prediction entropy metric, a perturbation entropy shift characterisation, a cross-view agreement analysis, an aleatoric/epistemic decomposition, and an empirical characterisation of override correctness grounded in the DRT learning dynamics. Empirical evaluation on synthetic workloads indicates that 78% of successful overrides concentrate in high-entropy regions (H>0.85 bits), and the entry gate ablation of Section 5.13 supports the interpretation that override correctness remains substantially higher in high-entropy regions even when the gate is removed, which is consistent with the interpretation that the entropy dependence is an intrinsic property of the prediction landscape.

Future directions include extending the preliminary SPEC CPU 2017 [22] evaluation to a complete set of industry standard benchmarks within full-system simulators [25] for end-to-end IPC evaluation, extending the entropy framework to multi-bit perturbation with learned importance weights, exploring Rényi entropy variants for improved sensitivity to tail events in the prediction distribution, and applying the counterfactual stability framework to other microarchitectural prediction structures such as value prediction and prefetching [26].

## Figures and Tables

**Table 1:** Comparison with representative TAGE family predictors along dimensions relevant to counterfactual stability modelling.

Predictor	Mixed History	Statistical	Counterfactual	Entropy	Selective	Information
	Views	Correction	Stability	Analysis	Override	Theoretic
L-TAGE [2]	Limited	No	No	No	No	No
GL-TAGE [6]	Yes	No	No	No	No	No
TAGE-SC-L [4,5]	Limited	Yes	No	No	No	No
MTAGE+SC [7]	Yes	Yes	No	No	No	No
Multiperspective Perceptron [8]	Yes	Yes	No	No	No	No
Mirror-TAGE (this work)	Yes	Yes	Yes	Yes	Yes	Yes

**Table 2:** Default predictor parameters.

Component	Parameter	Value
Global history	GHR length	64 bits
Base predictor	Base table size	512 entries
Tagged path	Tagged banks	4
Tagged path	History lengths	4, 8, 16, 32
Tagged path	Entries per tagged bank	256
Tagged path	Tag width	10 bits
Alternate choice logic	Use-alt table size	128 entries
Statistical corrector	Tables/history lengths	3/8, 16, 32
Statistical corrector	Entries per SC table	128
Statistical corrector	Activation threshold $\tau_{sc}$	5
Mirror path	Mirror tables/history lengths	3/4, 8, 16
Mirror path	Entries per mirror table	128
Reliability control	Consensus threshold $\tau_{c}$	3
Reliability control	Margin threshold $\tau_{m}$	2
Reliability control	Gap threshold $\tau_{g}$	2
Reliability control	High-confidence threshold $\tau_{high}$	9
Replay control	DRT size	128 entries
Replay control	DRT authorisation threshold $\tau_{d}$	2

**Table 3:** Main workload configurations.

Workload	Class Mix	Noise	Fragile	Total	Warmup	Measured
	(C0/C1/C2)	Rate	Intensity	Events		Events
B1	4/16/44	1.0%	2	120,000	30,000	90,000
B2	12/22/30	1.5%	2	120,000	30,000	90,000
B3	8/16/40	3.0%	3	120,000	30,000	90,000

**Table 4:** Main results across workloads (mean ± standard deviation, n=20 seeds).

Workload	Baseline	Mirror-TAGE	Absolute	*p* Value	Override	Override	95% CI
	Accuracy	Accuracy	Gain		Count	Correctness	(pp)
B1	78.9 ± 0.8%	79.7 ± 0.7%	0.82 pp	<0.001	2281	66.1%	[0.62, 1.01]
B2	79.3 ± 1.0%	80.3 ± 0.9%	1.05 pp	<0.001	2392	69.7%	[0.79, 1.30]
B3	77.0 ± 0.7%	77.9 ± 0.7%	0.88 pp	<0.001	2610	65.3%	[0.70, 1.06]

**Table 5:** Information entropy statistics on SAFE_TUNE_B1 (mean over twenty seeds, rounded to two significant figures).

Metric	Value
Mean local prediction entropy (all branches)	0.41 bits
Mean local entropy (override events only)	0.89 bits
Mean local entropy (successful overrides)	0.93 bits
Mean perturbation entropy shift $∆H^{\mathrm{agg}}$	0.23 bits
Cross-view agreement rate (consensus cases)	96%
Cross-view agreement rate (non-consensus cases)	59%
Fraction of overrides with H>0.85 bits	78%
Aleatoric entropy (Class 2 mean)	0.19 bits
Epistemic entropy (Class 2 mean)	0.65 bits

**Table 6:** Class-wise accuracy on SAFE_TUNE_B1.

Metric	Baseline	Mirror-TAGE	Gain
Class 0 accuracy (Biased)	98.6%	98.6%	0.02 pp
Class 1 accuracy (Short-Range)	88.2%	88.4%	0.2 pp
Class 2 accuracy (Context-Fragile)	73.7%	74.8%	1.1 pp

**Table 7:** Ablation study on SAFE_TUNE_B1.

Variant	Accuracy	Gain vs. Baseline	Override Count	Override Correctness
Baseline	78.9%	–	0	–
Mirror Only	79.6%	0.67 pp	3326	59.1%
Mirror + Reliability	79.7%	0.79 pp	2525	64.1%
Mirror + DRT	79.6%	0.74 pp	2772	61.9%
Full Mirror-TAGE	79.7%	0.82 pp	2281	66.1%

**Table 8:** Capacity sensitivity on SAFE_TUNE_B1.

Scale	Predictor	Accuracy	Storage Bits	Gain
Small	Baseline	76.8%	14,496	–
Small	Equal Budget Baseline	77.0%	15,200	0.28 pp
Small	Mirror-TAGE	77.4%	15,200	0.61 pp
Default	Baseline	78.9%	18,880	–
Default	Equal Budget Baseline	79.2%	20,288	0.28 pp
Default	Mirror-TAGE	79.7%	20,288	0.82 pp
Large	Baseline	82.8%	27,648	–
Large	Equal Budget Baseline	83.1%	30,464	0.32 pp
Large	Mirror-TAGE	83.5%	30,464	0.74 pp

**Table 9:** Entry gate ablation on SAFE_TUNE_B1 (mean over twenty seeds).

Metric	With Gate ($F_{t}$)	Without Gate
Total overrides	2281	5840
Overall override correctness	66.1%	54.2%
Fraction of overrides with H>0.85 bits	78%	41%
Override correctness at H>0.85 bits	69.3%	63.7%
Override correctness at H≤0.85 bits	52.1%	47.6%
Net accuracy gain	0.82 pp	0.24 pp

**Table 10:** Preliminary results on SPEC CPU 2017 traces (mean ± standard deviation, n=20 seeds).

Benchmark	Baseline	Mirror-TAGE	Absolute	Override	Override	Override
	Accuracy	Accuracy	Gain	Freq. (%)	Correctness	Count
perlbench	96.4 ± 0.1%	96.7 ± 0.1%	0.26 pp	1.4%	61%	∼1100
gcc	95.2 ± 0.1%	95.6 ± 0.1%	0.38 pp	1.9%	60%	∼1500
mcf	93.7 ± 0.2%	93.9 ± 0.1%	0.21 pp	1.6%	57%	∼1300
deepsjeng	97.8 ± 0.1%	97.9 ± 0.1%	0.04 pp	0.8%	55%	∼640
xalancbmk	94.9 ± 0.1%	95.2 ± 0.1%	0.26 pp	1.7%	59%	∼1400
x264	97.2 ± 0.1%	97.3 ± 0.1%	0.13 pp	1.1%	57%	∼880
Mean	95.9%	96.1%	0.21 pp	1.4%	58%	∼1100

## Data Availability

The original contributions presented in this study are included in the article. Further inquiries can be directed to the corresponding author.

## References

[1] Seznec A., Michaud P. (2006). A case for (partially) tagged geometric history length branch prediction. J. Instr. Level Parallelism.

[2] Seznec A. (2007). The L-TAGE branch predictor. J. Instr. Level Parallelism.

[3] Jiménez D.A., Lin C. (2001). Dynamic branch prediction with perceptrons. Proceedings of the 7th International Symposium on High-Performance Computer Architecture (HPCA), Monterrey, Mexico, 19–24 January 2001.

[4] Seznec A. TAGE-SC-L branch predictors. Proceedings of the 4th Championship Branch Prediction (CBP-4).

[5] Seznec A. TAGE-SC-L branch predictors again. Proceedings of the 5th Championship Branch Prediction (CBP-5).

[6] Ishii Y. Global-local combined branch history: The alternative way to improve TAGE branch predictor. Proceedings of the 4th Championship Branch Prediction (CBP-4).

[7] Seznec A. Exploring branch predictability limits with the MTAGE+SC predictor. Proceedings of the 5th Championship Branch Prediction (CBP-5).

[8] Jiménez D.A. Multiperspective perceptron predictor. Proceedings of the 5th Championship Branch Prediction (CBP-5).

[9] Jiménez D.A. (2005). Piecewise linear branch prediction. Proceedings of the 32nd Annual International Symposium on Computer Architecture (ISCA), Madison, WI, USA, 4–8 June 2005.

[10] Lin C.K., Tarsa S.J. (2019). Branch prediction is not a solved problem: Measurements, opportunities, and future directions. Proceedings of the 2019 IEEE International Symposium on Workload Characterization (IISWC), Orlando, FL, USA, 3–5 November 2019.

[11] Schall D., Sandberg A., Grot B. (2024). The last-level branch predictor. Proceedings of the 57th IEEE/ACM International Symposium on Microarchitecture (MICRO), Austin, TX, USA, 2–6 November 2024.

[12] Cover T.M., Thomas J.A. (2006). Elements of Information Theory.

[13] Desmet V., Eeckhout L., De Bosschere K. (2006). Improved composite confidence mechanisms for a perceptron branch predictor. J. Syst. Archit..

[14] Chen I.K., Coffey J.T., Mudge T.N. (1996). Analysis of branch prediction via data compression. Proceedings of the 7th International Conference on Architectural Support for Programming Languages and Operating Systems (ASPLOS), Cambridge, MA, USA, 1–5 October 1996.

[15] Zangeneh S., Pruett S., Lym S., Patt Y.N. (2020). BranchNet: A convolutional neural network to predict hard-to-predict branches. Proceedings of the 53rd Annual IEEE/ACM International Symposium on Microarchitecture (MICRO), Athens, Greece, 17–21 October 2020.

[16] Shannon C.E. (1948). A mathematical theory of communication. Bell Syst. Tech. J..

[17] Goldfeld Z., Polyanskiy Y. (2020). The information bottleneck problem and its applications in machine learning. IEEE J. Sel. Areas Inf. Theory.

[18] Saxe A.M., Bansal Y., Dapello J., Advani M., Kolchinsky A., Tracey B.D., Cox D.D. (2019). On the information bottleneck theory of deep learning. J. Stat. Mech. Theory Exp..

[19] Schreiber T. (2000). Measuring information transfer. Phys. Rev. Lett..

[20] Kendall A., Gal Y. (2017). What uncertainties do we need in Bayesian deep learning for computer vision?. Adv. Neural Inf. Process. Syst..

[21] Hüllermeier E., Waegeman W. (2021). Aleatoric and epistemic uncertainty in machine learning: An introduction to concepts and methods. Mach. Learn..

[22] Bucek J., Lange K.D., Kistowski J.v. (2018). SPEC CPU2017: Next-generation compute benchmark. Companion of the 2018 ACM/SPEC International Conference on Performance Engineering.

[23] Hennessy J.L., Patterson D.A. (2017). Computer Architecture: A Quantitative Approach.

[24] Akram A., Sawalha L. (2019). A survey of computer architecture simulation techniques and tools. IEEE Access.

[25] Gober N., Chacon G., Wang L., Gratz P.V., Jiménez D.A., Teran E., Pugsley S., Kim J. (2022). The championship simulator: Architectural simulation for education and competition. arXiv.

[26] Bera R., Kanellopoulos K., Nori A.V., Shahroodi T., Subramoney S., Mutlu O. (2021). Pythia: A customizable hardware prefetching framework using online reinforcement learning. Proceedings of the 54th IEEE/ACM International Symposium on Microarchitecture (MICRO), Virtual, 18–22 October 2021.

